# Quantifying Averted Disability-Adjusted Life Years as a Performance Indicator for Water Quality Interventions: A Review of Current Methodologies and Challenges

**DOI:** 10.3390/w10060744

**Published:** 2018-06-07

**Authors:** Darcy M. Anderson, Evan A. Thomas, Thomas F. Clasen

**Affiliations:** 1 Department of Environment Health, Rollins School of Public Health, Emory University, Atlanta, GA 30322, USA;; 2 Mortenson Center for Engineering in Developing Communities, University of Colorado at Boulder, Boulder, CO 80309, USA;

**Keywords:** water quality, disease burden, ADALYs, pay for performance, results based financing, monitoring

## Abstract

Sustainable access to safe drinking water protects against infectious disease and promotes overall health. Despite considerable progress toward increasing water access, safe water quality and reliable service delivery remain a challenge. Traditional financing strategies pay implementers based on inputs and activities, with minimal incentives for water quality monitoring and sustained service operation. Pay-for-performance offers an alternative financing strategy that delivers all or a portion of payment based on performance indicators of desired outputs or outcomes. A pay-for-performance approach in the water sector could quantify and incentivize health impact. Averted disability-adjusted life years (ADALYs) have been used as a performance indicator to measure the burden of disease averted due to environmental health interventions. Water-related disease burden can be measured for application as an ADALYs performance indicator following either comparative risk assessment or quantitative microbial risk assessment. Comparative risk assessment models disease burden using water source type as a proxy indicator of microbial water quality, while quantitative microbial risk assessment models disease burden using concentrations of indicator pathogens. This paper compares these risk assessment methodologies, and summarizes the limitations of applying these approaches toward quantifying ADALYs as a performance indicator for water quality interventions.

## 1. Background

Sustained access to a safe drinking water supply is essential for health. Safe drinking water reduces exposure to pathogens, decreasing morbidity and mortality from infectious disease [1]. Poor water quality is associated with a range of long-term health impacts, as repeated enteric infections during childhood can lead to malnutrition and permanent sequelae such as stunting and impaired cognitive development [2]. Insufficient water quantity is associated with an increased risk of dermatologic, ocular [3], and respiratory [4] infections. For a majority of households in the rural areas of low-income and middle-income countries, water must be collected from communal sources outside the home. In areas with limited or unreliable access, individuals may dedicate multiple hours per day to collecting water. Increased water collection times are associated with increases in child diarrhea, stunting, and mortality [5]. Water collection as a domestic chore has been shown to reduce time spent in school [6], and time dedicated to water collection could otherwise be used for leisure or income-generating activities [7].

The United Nations estimates that the water access target under the Millennium Development Goals (MDGs) was met five years ahead of schedule. While water access has increased substantially in recent decades, an estimated 663 million people globally still lack access to safe drinking water, and significant disparities persist both between and within countries [8]. Furthermore, the indicators for monitoring progress toward the MDG target fall short in measuring water safety and reliability, likely meaning that access estimates overrepresent the true proportion of the global population with reliable access to safe drinking water [9].

The MDGs measure access to safe and sustainable water using indicators created by the World Health Organization (WHO) and the United Nation Children’s Fund’s (UNICEF) Joint Monitoring Program (JMP). JMP indicators track service level and categorize sources as “improved” or “unimproved” as a proxy for water quality without directly measuring microbial safety [10], although the JMP itself has recognized that these indicators are poor proxies for microbial safety [11]. Water quality data from multiple countries suggest that more than a quarter of improved sources contain levels of fecal contamination that exceed WHO guidelines.

In addition to issues concerning water quality monitoring, high levels of periodic non-functionality and discontinuity of service across water sources have resulted in overestimates of access [12]. As water supply systems age, a lack of community or institutional capacity, gaps in operation and maintenance budgets, the inability of sources to meet growing demand, and a failure to monitor source performance all contribute to decreased functionality [13,14]. The resilience of water supply systems relies on ongoing performance monitoring and incentives for implementers to provide continuous, high-quality service [15]. Without the long-term monitoring of service delivery, measurements of water availability and the implied health benefits can be misleading. For example, from 1990 to 2015, access to improved water in rural sub-Saharan Africa apparently increased from 34% to 56% [8]. However, as many as a third of communal boreholes in sub-Saharan Africa may be non-functional at any given time, and many are never repaired [16].

Currently, a majority of aid projects are delivered with a near-term focus on implementation, monitoring, and maintenance over a limited number of years. Some projects may have funding dedicated for randomized controlled trials or other forms of impact evaluation, but this funding rarely extends to long-term monitoring over the intended life cycle of the intervention, and most rural communal sources are not regularly monitored for water quality or functionality in low-income and middle-income countries [17–19]. Although sustainability is being increasingly recognized as a critical element of water supply [17,20,21], many projects are still implemented without any clear definition of sustainability or explicit provisions to improve long-term functionality and continuity [17].

## 2. Incentivizing Health Gains over Infrastructure Coverage

### 2.1. Current Delivery Approaches

Since the 1980s, the dominant paradigm for rural water supply management has been community-based, in which the operation, maintenance, and collection of user fees are the responsibility of a local governing body. This paradigm grew out of the theory that national governments had insufficient resources to manage the highly dispersed network of rural water supplies and that local governance was more cost-effective [17]. Community-based management also appealed to bilateral aid and non-governmental donors, who were able to implement a project over the course of months to years, before shifting responsibilities to the community and relinquishing any control or responsibility for long-term operation and maintenance [15].

Community managed systems should, in theory, function with local labor and funding from user fees to support operation and maintenance [20]. However, multiple studies have shown that user fees for communal sources are often not regularly collected, leading to insufficient funds for routine operation and maintenance. Furthermore, appropriate replacement parts and skilled labor are not always reliably available, limiting the capacity for repairs [22–24].

In response to the shortcomings of community-managed models, a growing number of studies have criticized the current paradigm of water delivery as insufficient to ensure safe and sustainable access to water supplies, which are the prerequisites to genuine health impacts. This has increasingly resulted in calls for alternative financing and program delivery models that better align monitoring and incentive methodologies with intended long-term health impacts [15,20,25,26].

### 2.2. Pay-for-Performance Financing

Pay-for-performance, which is also called results-based financing, is one alternative financing scheme that addresses the challenges of traditional financing and program delivery and can better align the desired impact with a mix of funding mechanisms [26]. Its goal is to align payment incentives for implementers with the funder’s desired health or social impacts through making the delivery of payment or resources conditional on the achievement of particular performance indicators [15]. In recent years, many funders have increased requirements for accountability and transparency from program implementers [27], requiring more systematic evaluation and reporting of program results through results-based frameworks [28,29]. New interest in pay-for-performance and results-based frameworks stems from the theory that funding mechanisms can be used to improve implementers’ social performance through measuring impacts such as service quality, beneficiaries targeted, or program efficiency [15].

Traditional financing and program delivery models link funding to the completion of specific activities or provision of short-term outputs (e.g., the construction of a borehole), rather than longer-term outcomes (e.g., sustained, continuous community access to water) or impacts (e.g., reduced child mortality from diarrhea). Under such a system that emphasizes short-term outputs, incentives for monitoring health impacts over the long term or improving program delivery are minimal [30,31]. In contrast, under pay-for-performance financing, funders deliver all or some proportion of payment conditional upon implementers achieving pre-specified performance targets. Rather than incentivizing short-term outcomes, pay-for-performance instead incentivizes long-term indicators of sustained program outcomes and impacts. Funders and implementers agree to a contract that defines performance indicators and a methodology for determining payment based on the achievement of those performance targets, and implementers accept the financial risk of achieving those targets [15].

Randomized controlled trials have long been considered the gold standard for health impact evaluation. However, while strict requirements for the control of program delivery and adherence to pre-defined protocols lend valuable rigor to randomized controlled trial designs, they do not allow for corrections or improvements during program delivery, and conclusions from these evaluations may only be applied towards future interventions [15]. Pay-for-performance allows for continuous improvement to service delivery and greater autonomy and innovation among implementers, as the focus is on achieving specific objectives rather than the means by which those objectives are achieved [26]. Furthermore, the participatory design and management of water projects where funders and implementers agree on the roles, responsibilities, and objectives early in the process of project design can improve sustainable management practices [32].

Precise and transparent definitions of performance indicators and payment methodologies are critical for successful pay-for-performance financing. Clearly defined indicators and incentives help align risks and goals for all parties, so that when implementers meet established performance targets, both parties are satisfied, and project benefits are maximized for the target population [15]. Research has shown that pay-for-performance systems can lead to unintended distortions in project implementation if performance incentives are poorly defined or misaligned with project goals [33].

Performance indicators must be specific and measurable so that incentives may be delivered precisely and reliably [30,31]. Furthermore, indicators should be selected to measure outputs or outcomes that lie along the causal chain to achieving project objectives. Where the relationship between performance targets and desired outcomes is not robust, pay-for-performance financing may promote an inefficient or even counterproductive use of resources by project implementers [26].

A variety of mechanisms and implementations have demonstrated varying approaches to pay-for-performance financing. Within the water sector, current funding mechanisms primarily use short-term outputs and intermediate outcomes as performance indicators, such as the provision of infrastructure or sustained source functionality, rather than long-term estimates of health impacts. While health impacts are the ultimate goal, and intermediate outcomes do not necessarily imply long-term health improvements, extending monitoring efforts beyond immediate outputs has shown promise in improving long-term sustainability. A 2015 review identified 65 completed and ongoing water, sanitation, and hygiene (WASH) programs using pay-for-performance financing. A majority of these programs were small-scale, with a median number of beneficiaries of 142,810. Overall, 94% of programs were successful in meeting their target performance indicators, although comparisons of effectiveness of pay-for-performance versus conventionally financed programs are lacking [34]. Several examples of pay-for-performance programs within the water sector are reviewed here, and the specific objectives, performance indicators, and payment mechanisms of a subset of these examples are described in Table 1.

As of 2018, the World Bank has awarded nearly $20 billion United States dollars in pay-for-results financing contracts [35]. Among these, the Rural Water Supply and Sanitation program in the Red River Delta region of Vietnam is targeted to provide over three quarters of the total budget requirements as a pay-for-results mechanism [36,37]. However, the contracting approach of payment for functional services has conflicted with the upfront cost of infrastructure installation, and has resulted in third-party lending in order to meet performance targets [37].

Bilateral aid is increasingly incorporating pay-for-performance. In partnership with the Government of Indonesia, the Australian Agency for International Development’s *Water and Sanitation Hibah* program awarding payment based on verified functional connections of households to water and sanitation services increased access to water and sanitation services by approximately 470,000 people [38]. In Tanzania, the United Kingdom Department for International Development has invested £150 million in the water sector since 2014, including a £78.6 million payment-by-results program, with a focus on increasing water access and water point functionality. In this program, the Tanzania Ministry of Finance is paid for each new, and each continually functioning water point in the country. The Ministry of Finance is then responsible for transferring funding to local government offices. While promising, this model has limitations that increase the sample error, including infrequent third-party monitoring and a reliance on (non-independent) local government level reporting. The increase in risk to the contracting agency may also translate to a larger risk premium, and increased cost for the targeted outcomes [39].

Private foundations and social enterprises are also engaged in pay-for-performance contracting. The Rockefeller Foundation and Yunus Foundation have supported the Social Success Note that enables commercial investment in social businesses, which is repayable both financially and through demonstrated social impact [40]. On a local level, increased community willingness to pay for improved water services has been demonstrated on rural handpumps. The continuous monitoring of handpumps with remotely reporting cellular sensors resulted in increased water point functionality, and a five-fold increase in community willingness to pay for water services [41,42]. In several programs, carbon financing under a voluntary and United Nations-mediated mechanism has been applied to household water filters. Within these programs, the ongoing monitoring of household use of the water filters is required and tied proportionally to the carbon credit commodity earned and sold by the implementer. These programs have required only periodic survey audits, and would benefit from direct measures of water quality and health impact [43].

## 3. Measuring Disease Burden as a Performance Indicator

### 3.1. Health Impact Performance Indicators

Ideally, performance indicators are as closely aligned with programs’ final health goals as possible to minimize perverse incentives. Beyond the water sector, efforts to measure and incentivize health impact as a performance indicator for interventions targeting household environmental quality have recently been demonstrated in the improved cookstove sector. The Household Air Pollution Intervention Tool (HAPIT) models personal exposure to airborne particulate matter and the adoption of improved cookstoves within a population and generates health impact estimates [46]. The HAPIT model uses dose-response curves to estimate health impact in terms of the averted disability-adjusted life years (ADALYs or DALYs averted) attributable to project intervention. Incorporating the HAPIT dose-response model, the Gold Standard, a voluntary trading mechanism previously used exclusively for carbon finance, has created a “health credit” commodity that is measured in terms of ADALYs for generation and sale [47].

A similar ADALYs model could be applied to estimate health impacts within the water sector, using indicators of water quality to estimate averted disease burden as a performance indicator. In the case of water quality interventions, these health goals can be defined in terms of reductions in disease burden due to exposure to pathogens in unsafe water. Two distinct approaches exist for estimating water-related disease burden using water quality indicators: comparative risk assessment and quantitative risk assessment (QMRA). Comparative risk assessment uses service-level indicators defined by the JMP [10], and has been most commonly employed to estimate global water-related disease burden in low-income and middle-income countries, such as in the Global Burden of Disease studies [48–51]. Alternatively, QMRA uses microbial contamination indicators, following safety standards established in the WHO’s *Guidelines for Drinking-water Quality* [52] and has most commonly used to evaluate the safety of individual water supply and distribution systems [53].

Both comparative and quantitative microbial risk assessment may be applied to measuring ADALYs as a performance indicator for water quality interventions. Section 3.2 below presents an overview of applying risk assessment models to estimate ADALYs. The indicators and methodologies used under each risk assessment approach are critically reviewed in Sections 3.3 and 3.4, respectively. Subsequent sections address the limitations of applying either risk assessment approach to measuring water quality exposure, service delivery, and health outcomes beyond diarrheal disease.

### 3.2. Estimating Averted Disability-Adjusted Life Years

ADALYs are estimated by assessing disease burden in DALYs pre-intervention and post-intervention, and attributing reductions in burden to project intervention. The difference between pre-intervention and post-intervention DALY estimates is adjusted for the proportion of the target population exposed to unsafe water *(E)*, and the proportion using the intervention technology *(𝒰)* to estimate DALYs averted due to project implementation [47]:

$$\text{ADALYs}=(\text{DALY}s_{\text{pre}}-\text{DALY}s_{\text{post}})*E*𝒰$$

**Figure 1.**
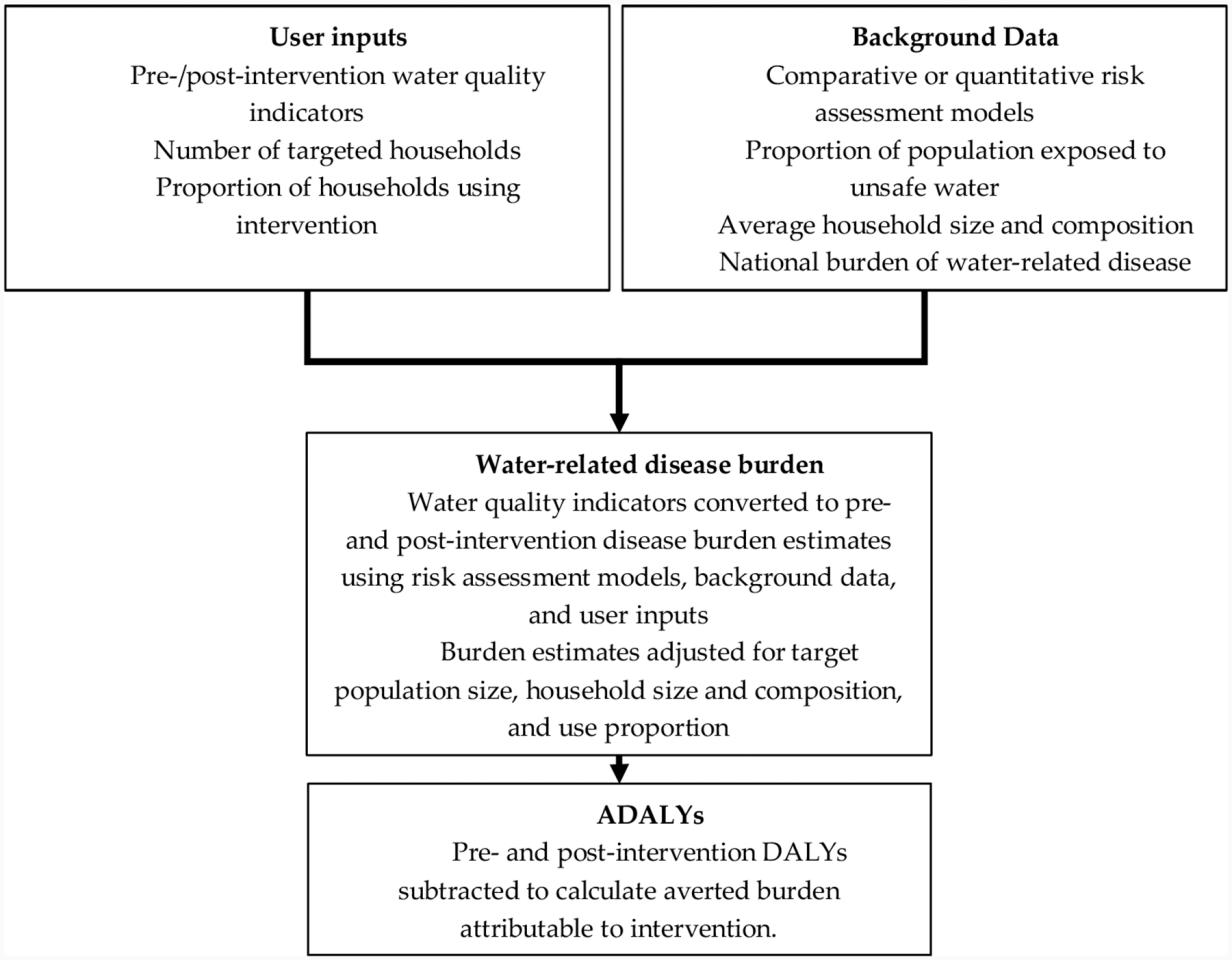
Schematic illustrating the process of estimating ADALYs using user-supplied and background data.

DALYs are calculated using project-specific pre-intervention and post-intervention water quality indicators with published risk assessment models. Unsafe water exposure and technology use are evaluated at the household level, and the overall total population health impacts are estimated using background demographic data on household size and composition. A schematic of the methodology for estimating ADALYs is illustrated in Figure 1.

Each risk assessment methodology yields a burden estimate in DALYs associated with water quality. ADALYs are not disease-specific, and are therefore well-suited to serve as a performance indicator to measure the aggregate health impacts of a single intervention and allow for broad comparability and applicability across interventions. Although ADALYs can combine health impacts across multiple conditions, currently background data and evidence supporting both risk assessment models are limited to estimating diarrheal disease burden only and are therefore expected to underestimate the overall burden of water-related disease [54,55]. The precise burden of all water-related disease is difficult to estimate. A variety of adverse health conditions related to microbial and chemical contamination, as well as poor access to sufficient water quantity, all contribute to the total burden of water-related disease, and existing data are insufficient to quantify their precise burden [56,57]. However, diarrheal diseases are considered to form the majority of water-related disease burden, and approximately 94% of diarrheal burden is attributable to poor WASH conditions [57].

Modeling health impacts as ADALYs through cross-sectional assessments of environmental exposure indicators allows for more timely and cost-effective evaluation of health interventions, rather than measuring health outcomes directly through longitudinal study designs [46]. While other impact evaluation methodologies, such those that use non-intervention areas to establish a counterfactual, may provide more rigorous estimates of intervention impact, the pre/post comparison employed in an ADALYs approach allows for more feasible rapid and ongoing evaluation suited to long-term pay-for-performance financing and funders who may have limited resources for rigorous impact evaluation beyond the intervention area.

### 3.3. Quantifying ADALYs through Comparative Risk Assessment

Comparative risk assessment quantifies disease burden by evaluating the risk of current exposure scenarios relative to an ideal hypothetical minimum risk scenario [58]. Relative risk assessments are derived from systematic reviews of epidemiologic studies that evaluate risk associated with different water services levels. Relative risks are used to calculate the fraction of the total diarrheal disease burden attributable water quality. Total water-related disease burden estimates are obtained by multiplying the national disease burden in DALYs by the percent fraction that is attributable to poor water quality.

Epidemiologic studies, and consequently systematic reviews, most commonly define service levels using indicators of improved versus unimproved sources following the WHO and UNICEF’s Joint Monitoring Program. Some systematic reviews have further refined JMP categorizations by separating piped household connections from other improved community sources or by considering additional improvements in water quality that are associated with point-of-use household treatment and safe storage [58–63]. Compared to the collection of water samples and microbial testing, JMP and other service-level indicators serve as a rapid and relatively simple water quality monitoring tool. However, without testing microbial safety, JMP indicators may misclassify improved water sources as safe despite the presence of fecal contamination [64], or vice versa.

Comparative risk assessment approaches rely on the robustness of relative risk estimates from systematic reviews, which consistently evaluate the quality of evidence to date as poor [58,60,62,65]. Estimates of WASH-related disease burden vary significantly depending on the methodologies and effect estimates used, and effect estimates show considerable heterogeneity across meta-analyses. Central to this debate are questions surrounding the rigor of published studies. The body of literature on water quality contains a large number of observational studies and non-randomized trials. Inclusion of these studies increases the number of comparisons available for analysis, but may bias effect estimates due to their lower methodological quality [66].

Due to the nature of intervention delivery for community-based water quality improvement, blinding is rarely ethically viable. However, a lack of blinding combined with self-reported diarrhea outcomes raises concerns over reporting bias from participants and study personnel [67]. While pooled effect estimates of blinded and non-blinded studies suggest that point-of-use water quality interventions reduce diarrhea, blinded trials have failed to demonstrate any effect [62]. Adjustment for non-blinding is possible, and results in effect estimates that are smaller but typically remain significant [58,61,62]. However, non-blinding adjustments should be interpreted with caution, as adjustments are derived from clinical studies, which may have limited applicability to environmental interventions [68].

In addition to the bias surrounding a lack of blinding, low adherence and adoption, particularly for point-of-use treatment, remain a challenge. Research has shown that even occasional exposure to untreated drinking water can vitiate the protective effects of consistent water treatment in the home [69]. Acceptability and adoption of point-of-use household treatment has been demonstrated to be low in multiple settings and often declines over time [70–77]. Overall, the lack of reporting on measures of adoption and compliance limits the possibility to adjust for associated biases [58,66]. Some reviews have attempted to attempt to control for low adherence—for example, Wolf et al. [58] excluded studies with less than 20% adoption, but this relatively low exclusion threshold is unlikely to adequately control for low adoption across included studies.

### 3.4. Quantifying ADALYs through Quantitative Microbial Risk Assessment

QMRA quantifies disease burden using dose-response models to estimate the disease risk at varying concentrations of waterborne microbial indicator species [53]. Indicator species concentrations are used to estimate the dose of pathogens ingested, which can be converted into the risk of diarrheal disease using dose-response curves. Background data on infectivity constants and the dose-response relationships used in QMRA models are drawn from primarily from clinical challenge studies. Disease burden is quantified using disease risk estimates and per-case disease burden weights in DALYs [52,78].

The WHO’s *Guidelines for Drinking-water Quality* outline the microbial performance indicators for water systems based on disease risk relative to concentrations of pathogenic indicator species [52]. Testing for all of the possible waterborne pathogens is infeasible, so monitoring a subset of indicator species to represent the overall disease risk is done under the rationale that the adequate control of indicator species will also result in the adequate control of other pathogens [52]. However, the distribution and concentration of indicator species relative to other pathogenic species differs across environmental contexts [79]. The fraction of diarrhea attributable to the four most common etiologic agents has been shown to represent less than half of all of the cases across multiple countries [80]. Monitoring and disease burden estimates derived using reference pathogens fail to capture the disease burden contributed by non-indicator species [79].

QMRA was developed based on a chemical risk assessment paradigm [53]. However, individual responses vary more across microbial exposures than chemical exposures, as virulence depends on characteristics of both the pathogen itself and the immune response of the host [81]. Constants for infectivity and virulence derived primarily from challenge studies of adult populations in high-income countries are applied universally across other populations [53,78]. In low-income and middle-income countries, where individuals may have compromised nutritional status or face additional immune challenges, responses to infection may be more severe [52]. Additionally, evidence from challenge studies in adults is likely insufficient to accurately estimate the burden of disease in children [82], who are at greater risk for infection and bear the majority of the global diarrhea burden [83]. QMRA models are also likely insufficient for other vulnerable populations, including pregnant women, the elderly, and other immunocompromised individuals [84,85].

In addition to variation in host response, virulence also varies by pathogen strain and the ingested dose. Different strains of *Cryptosporidium parvum*, for example, have been shown to have a median infectious dose ranging from 9 oocysts to 1042 oocysts, depending on the strain ingested [86]. Systematic reviews have shown that significant variation in infectivity constants is common across a wide variety of foodborne and waterborne pathogens, often varying by several orders of magnitude across different strains of the same species [87]. Furthermore, statistical power at low doses is often limited, as large sample sizes are need to define the lower bound of the dose-response curve, but the logistics and expense of challenge studies often prohibit large samples. Wide variation across infectivity constants results in a high degree of uncertainty surrounding dose-response relationships and final disease burden estimates [85].

### 3.5. Challenges of Exposure Assessment

Exposure assessment remains a challenge under both QMRA and comparative risk assessment methodologies. In the absence of comprehensive WASH, populations receiving only water quality interventions are expected to be protected from pathogens transmitted through the direct consumption of contaminated fluids, but remain at risk through transmission pathways related to poor sanitation and hygiene [65,88]. Fecal–oral transmission of diarrheal disease has been commonly conceptualized using the “F-diagram,” illustrating the transmission of fecal pathogens through fluids, fingers, food, fields, and flies [89]. Interventions to improve water quality primarily target pathogens transmitted through fluids, while interventions to improve sanitation and hygiene target the remaining pathways [88]. Where WASH conditions are poor, disease risk posed by sanitation and hygiene pathways may overwhelm any potential benefits of water quality interventions, regardless of treatment efficacy [66,90]. Heterogeneity in effect estimates across studies is likely in part due to varying environmental contexts and the relative importance of multiple transmission pathways in different settings [90].

Comparative risk assessment methods rely on field studies of water quality interventions conducted in the context of existing external sanitation and hygiene conditions. Compared to clinical challenge studies and QMRA, field studies offer the potential for a more holistic assessment of diarrheal risk under actual exposure conditions [85]. Studies of water quality interventions delivered in community-based settings by design include the effects of transmission through other non-targeted pathways [90], and disease burden estimates derived from epidemiologic studies therefore reflect the aggregate risk of diarrhea in the context of other unmitigated exposure routes. In contrast, QMRA models consider the transmission of infectious agents only from a single point source [53]. While QMRA directly measures water quality, and therefore provides a more accurate assessment of the microbial safety of individual sources than JMP classifications [85], QMRA models do not capture the effects of external pathways, including transmission from person-to-person or through alternative environmental pathways or water sources [53].

### 3.6. Challenges of Service Delivery Measurement

Measurements of coverage and the use of water and sanitation services are relevant inputs to an ADALY model. Coverage and use of household-level interventions are typically assessed using surveys and observations. However, a variety of studies have noted a poor correlation between self-reported and observed practices when evaluating the use of environmental interventions [70,72,91], and bias in self-reported outcomes is particularly likely when self-reported behaviors are perceived to be desired by evaluators or the wider community. The process of data collection itself can impact behavior, as individuals change their practices in response to the knowledge that they are being monitored, which is a phenomenon known as reactivity or the Hawthorne effect [92]. Reactivity has been demonstrated by trials that have used structured observation as an alternative to self-reported behavior outcomes [93]. In some cases, sanitary surveys have been presented as tools for predicting microbial contamination [94]. However, sanitation inspections may have limitations as a method for identifying contaminated drinking water sources. In several recent studies, sanitary surveys were demonstrated as poor predictors of microbial contamination in drinking water [95,96]. The application of new monitoring technologies to water interventions may allow more cost-effective and measurable results [97]. Remote sensing technologies, via satellite assets and in situ sensors, have been developed and applied to monitoring water supply interventions [98]. A variety of technologies such as water quality sensors and flow meters may be applied to evaluate service quality and use practices, and data can be collected either via satellite, cellular, or wifi networks, or through manual collection by local enumerators. Advances in remote sensing technology have the potential to improve the frequency and validity of measurements, while minimizing reactivity and improving the accuracy of ADALYs estimates [97].

### 3.7. Evaluating Health Impacts beyond Diarrheal Disease

Both risk assessment methodologies ignore the possible effects of water quality interventions on water quantity and access. While point-of-use treatment may be expected to have minimal impact on water access, infrastructure improvements, such as house connections or the construction of new improved sources that reduce collection distance, will provide a range of health and well-being improvements beyond water quality alone. For example, water collection times of greater than 5 min are expected to reduce the amount of water used for personal and domestic hygiene [99], and research has shown that greater distances to a water source and longer collection times are associated with adverse child health outcomes [5]. Some studies have evaluated non-infectious health outcomes associated with water access, such as the caloric burden of water fetching [100], although non-infectious outcomes have been historically excluded from disease burden estimates. Other related outcomes, such as the time savings associated with water access [101], are relevant to the overall improvements in well-being associated with water but are rarely considered when evaluating health impacts. Changes in non-infectious and non-health outcomes are challenging to quantify in an ADALYs model but may represent significant improvements in well-being where interventions simultaneously impact water quality and quantity.

## 4. Discussion

Pay-for-performance financing has been used to incentivize long-term monitoring and the continued operation and maintenance of water supplies to improve service quality and sustainability. Models and proxy indicators may be used to estimate health improvements, but performance indicators and methodologies for determining payment must be specific, well defined, and robustly linked to desired health outcomes to avoid creating perverse incentives [26].

Pay-for-performance is gaining traction among funders and development agencies across a wide variety health and social programs as a method of increasing accountability and improving service delivery [27]. Within WASH interventions, recent efforts have attempted to link the disbursement of foreign aid payments to regular monitoring and quality indicators for WASH services [102]. ADALY estimates derived from environmental water quality indicators and risk assessment methodologies are an alternative metric that can be used to measure the aggregate disease burden averted as a performance indicator for health improvements from water quality interventions.

Existing water quality indicators and risk assessment methodologies to calculate water quality-related disease burden have a variety of limitations. Water quality indicators of improved/unimproved under the JMP rely on source type as a proxy for safety, which is known to correlate poorly with actual microbial risk [64]. ADALYs estimates calculated using JMP indicators will overestimate health benefits where the post-intervention quality of improved sources is unsafe. Revisions to JMP indicators have the potential to reduce uncertainty in comparative risk assessment models through the direct measurement of microbial safety.

JMP indicators under the Sustainable Development Goals do aim to include microbial safety in evaluating safely managed water systems [103]. New classifications for drinking water under the SDGs define the highest level of drinking water supply as “safely managed”, where the source is “located on the premises, available when needed, and free of fecal and priority contamination” [103]. A shift from the binary improved/unimproved classification to consider safe management and microbial contamination will likely change how epidemiologic studies of water quality interventions measure exposure. However, at present, consensus on how these new indicators will be measured is lacking, and how microbial safety monitoring will be incorporated into existing monitoring efforts remains unclear [104].

Microbial water quality indicators quantify concentrations of specific reference pathogens as a direct measure of disease risk. However, as etiologic agents of diarrhea vary significantly across different environmental contexts [80], monitoring only a select number of indicator species is unlikely to capture the entirety of diarrhea burden where etiologic agents vary widely and are not specifically measured within the suite of indicator species. ADALYs estimates using microbial indicators may prove more accurate where the etiologic agents responsible for a majority of the local burden of diarrhea can be identified, and indicator species are specifically selected to reflect the most salient etiologic agents in the local context.

Variation in both host response and the virulence of diarrhea pathogens makes a universal application of QMRA difficult. Challenge studies of healthy adult volunteers from high-income countries likely have limited applicability toward other populations in low-income and middle-income settings. Children, especially those facing malnourishment or additional immune challenges in poor WASH conditions, may likely have considerably different responses to microbial contamination [82]. This limitation is particularly relevant as the majority of diarrhea burden occurs in children [83]. While ethical considerations prevent challenge studies on children and other vulnerable populations, disaggregated data from outbreak investigations and other natural experiments can help to improve understanding of differential responses to exposure among these populations and improve disease burden estimates [82].

Exposure assessment related to the consumption of unsafe drinking water remains a challenge across both risk assessment approaches. Even where drinking water is free of contamination, individuals remain at risk of disease transmission related to poor sanitation and hygiene conditions [90]. QMRA models ignore the contribution of disease risk through non-water pathways [53], while comparative risk assessment models rely on epidemiologic studies that account for the contribution of non-water pathways, but cannot be reliably generalized to contexts where sanitation and hygiene conditions differ significantly [85]. The wide variation across the epidemiologic studies of water quality interventions is likely driven in part by the relative importance of these alternative pathways, although the precise relationship between different transmission pathways and their contribution to the total WASH-related disease burden remains poorly understood [90]. Further research to understand the relative importance and relationships of different components of WASH is needed to better quantify the effects of interventions in different contexts. Ultimately, the ADALYs models may need to account for the effects of local sanitation and hygiene conditions to accurately quantify the effects of water quality interventions.

ADALYs models under both risk assessment approaches fail to account for infectious disease burden beyond diarrhea or the effects of water quantity. Previous estimates of WASH-related global disease burden have applied the same relative risk estimates from systematic reviews of diarrheal disease studies to estimate the disease burden for other fecal–orally transmitted diseases such as typhoid and salmonella, although there is no empirical evidence to support this assumption [51]. Additional research on the impacts of water quality on other health outcomes would allow for more accurate estimates of total water-related disease burden in ADALYs models.

The current evidence base also does not allow ADALY models to account for a variety of infectious and non-infectious health and well-being outcomes associated with water quantity. QMRA models do not account for water quantity, and comparative risk assessment models only rudimentarily control for the effects of water access by considering sources requiring more than a 30-min round trip for collection time as unimproved [58]. However, hygiene activities are expected to be reduced, even where collection time exceeds five minutes in total [99]. Water quantity also provides non-health benefits, such as reduced time spent collecting water, that are difficult to quantify in ADALYs estimates, but contribute to overall well-being and warrant consideration when delivering water quality interventions.

The limitations of current indicators and risk assessment methodologies pose significant challenges for quantifying ADALYs as a performance indicator. The evidence base underpinning QMRA and comparative risk assessment models suffers from poor methodological quality, a lack of generalizability across populations in low-income and middle-income settings, and limited understanding of the relationships between WASH-related transmission pathways. However, pay-for-performance financing does not necessarily require a highly precise estimation of averted disease burden, so long as the methodologies for determining payment are clearly defined and understood by all of the stakeholders, and the performance indicators are well aligned with project goals.

The sample cost and cost-effectiveness of alternative measurement technologies and methods including instrumentation and regular water quality testing, when compared with surveys, has not been well-established. Similarly, while a growing number of projects are employing pay-for-performance financing, a rigorous evaluation of the impacts of financing strategies on indicators of sustainable service delivery is limited [34]. While these approaches have demonstrated improved data quality, future work should attempt to create statistical protocols and compare sampling costs and pay-for-performance transactional costs against the status quo in terms of cost per volume of clean water, or duration of a reliable water service.

ADALYs models can be revised as new evidence is generated. Particularly in the post-2015 development era, the SDGs will change standards for water quality indicators and monitoring, and new evidence will be incorporated to change existing methodologies for quantifying water-related disease burden. As the methodological quality of WASH literature and understanding of WASH-related disease transmission pathways improves, the methodologies for estimating ADALYs can be modified and updated to address current limitations.

## Figures and Tables

**Table 1.** Pay-for-performance water programs and their objectives, select performance indicators, and associated payment mechanisms.

Program	Objective(s)	Performance Indicators	Payment Mechanism
World Bank-funded *Results-Based Rural Water and Sanitation Under The National Target Program* in the Red River Delta of Vietnam [36,44]	To improve hygiene behavior, as well as increase and sustain access to rural sanitation and water supply in rural areas in the participating provinces	Number of new or rehabilitated functioning water supply connections to households in the participating provinces Number of households in participating provinces with sustainable water systems	Payments disbursed annually as a proportion of the indicator target achieved, with independent verification of indicators
United Kingdom Department for International Development-funded *Water, Sanitation*, *and Hygiene Results Programme* in 12 countries in sub-Saharan Africa and South Asia [38]	To bring equitable and sustainable water and sanitation services and hygiene practices to 4.5 million people in 12 countries, and thus to improve health by reducing diarrhea morbidity and child mortality	Number of people with access to water facilities Number of people using water facilities	Payments disbursed annually as a proportion of the indicator target achieved, with the independent verification of indicators
Australian Department for Foreign Affairs and Trade-funded *Water and Sanitation Hibah* in Indonesia [45]	To increase local government investment in water infrastructure, with an emphasis on low-income populations, and to improve the governance of the water sector at the local government level by increasing their accountability to adhere to an agreed water investment program and a level of incremental improvements to services	Number of household water connections verified for the following criteria: (a) the technical functionality of the connection, (b) low-income category of the household, and (c) two months of paid water bill	Local governments requested audits of eligible connections from central government, funding disbursed to local government per eligible connection upon verification
